# Waiting for the biopsy, finding goodbye

**DOI:** 10.1017/S1478951525100515

**Published:** 2025-09-11

**Authors:** Rafaella Cristina Curatolo, João Carlos Geber-Júnior

**Affiliations:** 1 Faculty of Health Sciences, Instituto Sirio-Libanes de Ensino e Pesquisa, São Paulo, Brazil; 2 Centro de Atendimento de Intercorrências Oncológicas (CAIO), Instituto do Câncer do Estado de São Paulo, Hospital das Clínicas da Faculdade de Medicina da, Universidade de Sao Paulo, São Paulo, Brazil

In August 2023, I was working in a public hospital in São Paulo, a reference center for palliative care. That month, I encountered a patient whose final hours revealed not only the fragility of life, but the depth of human connection when answers are delayed, and time becomes sacred.

Palliative care often means walking alongside those for whom curative options are no longer available. We offer presence, not certainty; support, not resolution. That day, I was called by a colleague to assist in communicating bad news to a man likely facing a terminal illness. He was around 50 years, experiencing severe respiratory failure, with a presumptive diagnosis of lung cancer. The biopsy result was still pending.

Despite his declining state, he held onto a single hope: to speak with his wife. As she was on her way, he called her, only to be met with the unexpected voice of his 5-year-old son, who told him how much he missed him. Tears welled up in the patient’s eyes, but he held them back – perhaps trying to protect his family from the weight of his vulnerability.

When we explained the need for intubation, he asked,

“Will you wake me up to tell me the biopsy result?”

“Only if it’s medically possible,” we answered.

He followed with a second plea: “Can I wait for my wife?”

We agreed.

After his transfer to the ICU, his mother, stepfather, and wife arrived. They were unaware of the transfer or the change in his condition. ICU policy allowed only one visitor at a time. His stepfather stayed behind. That quiet exclusion unsettled me.

Moved by the family’s distress, I intervened – requesting flexibility from the team. Bureaucratic resistance emerged, but I navigated around it gently. Without explicit permission, I allowed all three to be by his side.

His wife, standing in the corridor, wept: “We’ve been together for 35 years, since we were teenagers. My oldest son won’t stop crying. The youngest has a sore throat and refuses to go to the doctor – he says he won’t come home, just like his father. Why did the biopsy take so long? Why wasn’t his surgery scheduled?”

These questions bore emotional urgency, not clinical inquiry. They weren’t looking for answers, but for recognition. For someone to witness their fear.

As their brief visit ended, I stood by his bedside. The patient turned to me and asked,

“Do you think I’ll make it?”

I couldn’t lie.

“I don’t know if you will,” I said. “But we’ll be here, caring for you as best we can.

You’re not alone.”

He smiled faintly.

“Thank you, miss.”

He died that night.

He never heard the biopsy result. He died in an ICU he feared, in a system too slow to meet his questions. But because we allowed space for presence – his stepfather’s hand, his wife’s final kiss – he died accompanied, not forgotten.

To work in end-of-life care is to accompany what cannot be named. It is to witness sacred human bonds in their final form. Not all suffering can be measured, and not all care can be charted.

Illness fractures the continuity of the self. It interrupts the assumption of tomorrow. It exposes the body’s limitations and summons unfamiliar emotions – anger, despair, dependency. As Moretto writes, “The greatest suffering for any neurotic is the reenactment of their constitutive loss: they cannot tolerate losing anything” (Moretto 2019). The body becomes unfamiliar. “What body is this? I am no longer myself.”

In palliative care, we must distinguish clinical depression from existential grief. When a 55-year-old patient with metastatic ovarian cancer told her team she no longer wished to continue chemotherapy, her physician and husband questioned whether she was depressed. But perhaps her decision wasn’t pathological – it was lucid. The discomfort professionals often feel in such moments is less about diagnosis and more about confronting finitude.

As clinicians, we sometimes pathologize what we cannot fix. But not all despair is disordered. Sometimes, it is simply mourning – preemptive, quiet, dignified.

To intervene meaningfully, communication must carry resonance. Freud’s concept of transference reminds us: the meaning of words depends not only on content, but on who speaks them and how. In one of our training simulations, a man lashed out during his wife’s intubation. His outburst wasn’t resistance – it was grief in search of containment. “I didn’t feel supported,” he later said. “That’s why I couldn’t listen.”

Without emotional resonance, information falls flat. Netto notes that transferential dynamics can trigger fatigue, irritation, or helplessness in clinicians (Netto 2022). But these responses must be recognized, not denied. They are part of the clinical encounter. They shape how we show up for others – and for ourselves.

This narrative aligns with growing evidence that narrative-based interventions enhance empathy and emotional containment in palliative settings (Bingley et al. 2008; Laskow et al. 2019). In the ICU, integration of palliative approaches reduces moral distress and facilitates family-centered care (Palliative Care in the Intensive Care Unit 2023). Ultimately, effective end-of-life care is less about technical mastery and more about emotional presence (Engel et al. 2023).

To accompany a dying person is not to fix their condition, but to bear witness to their story. Sometimes, the greatest act of care is not in the cure, but in allowing someone to say goodbye.
